# ﻿*Aristolochiazhuhaiensis*, a self-supporting new species of Aristolochiaceae from Guangdong, China and notes on *Aristolochiathwaitesii*

**DOI:** 10.3897/phytokeys.254.139616

**Published:** 2025-03-21

**Authors:** Yi-Fan Wang, Zi-Rui Guo, Sven Landrein, Joyce G. Onyenedum, Shuai Liao

**Affiliations:** 1 Department of Environmental Studies, New York University, New York 10012, USA New York University New York United States of America; 2 Suzhou Lianhelvyou Ecological Agriculture Development Co., LTD, Suzhou 215026, Jiangsu, China Suzhou Lianhelvyou Ecological Agriculture Development Co., LTD Suzhou China; 3 Kadoorie Farm and Botanic Garden (KFBG) Corporation, Hong Kong S.A.R., China Kadoorie Farm and Botanic Garden Hong Kong Hong Kong; 4 South China Botanical Garden, Chinese Academy of Sciences, Guangzhou 510650, Guangdong, China Chinese Academy of Sciences Guangzhou China

**Keywords:** *Aristolochia* subg. *Siphisia*, conservation status, fieldwork, morphology, taxo­nomy

## Abstract

A self-supporting new species of Aristolochiaceae, *Aristolochiazhuhaiensis* Y.Fan Wang & Z.R.Guo, is described and illustrated from Zhuhai City, Guangdong Province, China. Morphologically, this species exhibits vegetative characters, including leaf blade shape and growth form, that are similar to *Aristolochiathwaitesii* Hook., the only other known taxon in Aristolochiasubg.Siphisia in Asia that is non-twining and self-supporting, native to southern Guangdong, particularly the Hong Kong Special Administrative Region. However, *A.zhuhaiensis* can be readily distinguished from *A.thwaitesii* by its unique floral morphology. The new species features a campanulate to funnel-shaped calyx limb densely covered with porcelain-white granular pustules. In contrast, *A.thwaitesii* has an elongated, concave tubular calyx limb with an adaxial surface that transitions from purple to pale yellow or white towards the apex, densely covered with recurved fleshy prickles. *Aristolochiazhuhaiensis* is endemic to Zhuhai City and has not been observed elsewhere. Detailed illustrations, photographic plates, a distribution map, a digitized holotype voucher specimen, a comparative table, and an identification key are provided to facilitate the differentiation of *A.zhuhaiensis* from *A.thwaitesii*.

## ﻿Introduction

*Aristolochia* L., the largest genus in Aristolochiaceae with more than 600 species distributed across tropical, subtropical, and temperate regions (Huber 1993; Neinhuis et al. 2005; Wanke et al. 2006), includes three recognized monophyletic subgenera: *Aristolochia*, *Pararistolochia* (Hutch. & Dalziel) Schmidt, and *Siphisia* (Duch.) Schmidt (González and Stevenson 2002; Wanke et al. 2006; Buchwalder et al. 2014; Ohi-Toma and Murata 2016). Subgenus Siphisia, notable for its morphological distinctiveness—strongly curved perianth, three-lobed gynostemium, and paired anthers (González and Stevenson 2000; 2002)—and high species diversity in the Indochina Peninsula and southeastern Himalayas, has been a focal point of taxonomic debate (Pfeifer 1966; González 1999; Huang et al. 2003; Ohi-Toma et al. 2006; Wanke et al. 2006; González et al. 2014). While systematic and molecular studies have consistently supported its monophyly within *Aristolochiasensu lato* (González and Stevenson 2002; Wanke et al. 2006), Zhu et al. (2019) conducted new phylogenetic analyses using more comprehensive genetic datasets and proposed its reinstatement to genus rank as *Isotrema* Raf., based on infra- and inter-genic markers. Since its publication, this treatment has been adopted by some scholars, primarily in new species descriptions (Li et al. 2019; Cai et al. 2020; Huang et al. 2022), contrasting with the prevailing *Aristolochiasensu lato* framework used in broader botanical studies (Phan et al. 2021; Van Do et al. 2021a; Van Do et al. 2021b). Here, while acknowledging the valid arguments for both perspectives, we adhere to the *Aristolochiasensu lato* framework, which aligns with long-standing taxonomic convention and robustly integrates both morphological and molecular evidence.

In *Aristolochia* s.l., the majority of species exhibit a climbing habit, either as herbaceous climbers or woody lianas (Wagner et al. 2014; Trueba et al. 2015). Wagner et al. (2014) demonstrated that the climbing habit, prevalent across most species in the genus, is a derived trait that evolved from herbaceous or shrub-like ancestors and likely facilitated the rapid diversification of Aristolochia s.s. and subg. Siphisia. Within subg. Siphisia, the evolution of shrub-like traits in certain species has been proposed to originate from climbing *Aristolochia* ancestors. Wagner et al. (2012) developed two hypotheses to explain the emergence of these traits, both based on the premise that climbing is the plesiomorphic state for subg. Siphisia. Notably, all non-climbing members of subg. Siphisia included in these studies were restricted to North and Central America.

In Asia, nearly all Aristolochiasubg.Siphisia species are climbers, with one notable exception: *Aristolochiathwaitesii* Hook. This species, previously the only documented self-supporting subshrub of Asian subg. Siphisia, is primarily found in coastal regions of Hong Kong SAR, China (Tutcher 1905; Dunn and Tutcher 1912; Barringer 1993; Xia 2007), and has also been reported in neighboring Guangdong cities such as Jiangmen and Zhuhai (Ma 1989; Huang et al. 2003). However, upon reviewing related literature and examining available voucher specimens of *A.thwaitesii*, we found that nearly all were collected from Hong Kong, with only two vegetative specimens from Guangdong (IBSC0127924 & SYS00159983). Beyond this, no fertile specimens or living collections from mainland China exist.

During a 2023 late spring expedition in Doumen District, Zhuhai City, as part of a traditional Chinese medicine survey, we discovered an *Aristolochia* species that is an erect non-climbing subshrub with leaf morphology very similar to *A.thwaitesii*. The plants have been collected and cultivated by locals for their dried roots, harvested for medicinal purposes. Based on its vegetative characteristics, we tentatively identified it as *A.thwaitesii* and collected samples for ex situ conservation, as this species is listed as Vulnerable under IUCN criteria A2c (China Plant Specialist Group 2019).

In late January 2024, the cultivated plants began to flower, revealing floral morphological features significantly different from *Aristolochiathwaitesii*. These differences, when compared to original descriptions and herbarium specimens, suggested it could potentially be a new species. Subsequent fieldwork from February to April across multiple locations in Zhuhai City uncovered several flowering populations of this *Aristolochia* species, all with consistent floral features matching those of the specimens we collected in 2023 from Doumen District and now under cultivation.

This unknown species is morphologically similar to *A.thwaitesii* in terms of plant habit, vegetative traits, and its connate geniculate perianth, but it is readily distinctive due to its floral morphology. It has a campaniform-infundibuliform calyx limb densely covered with porcelain-white granular pustules, whereas *A.thwaitesii* has an elongated concave tubular calyx limb with an adaxial surface transitioning from purple to pale yellow or white toward the calyx limb apex, fully covered with recurved fleshy prickles. This unknown species has been found exclusively in coastal dwarf forests situated in hilly areas, Zhuhai City.

Based on these findings, this study concludes that the newly discovered *Aristolochia* represents a new species, which is described and illustrated herein.

## ﻿Material and methods

The specimens examined in this study were collected from Doumen District, Zhuhai, Guangdong Province, China, and deposited at the Herbarium of the South China Botanical Garden (IBSC), Chinese Academy of Sciences. Morphological comparisons, including flower shape, color, and longitudinal sections of floral organs, were conducted against the original descriptions and voucher specimens of *Aristolochiathwaitesii*.

The conservation status assessment followed the IUCN Red List Categories and Criteria (IUCN 2012) and the latest Guidelines for Using the IUCN Red List Categories and Criteria (IUCN 2024). The Extent of Occurrence (EOO) and Area of Occupancy (AOO) were calculated using GeoCAT (Geospatial Conservation Assessment Tool) (Bachman et al. 2011), incorporating field data, ecological literature, and known distribution records to evaluate the species’ range and potential threats.

## ﻿Taxonomy treatment

### ﻿ Aristolochia zhuhaiensis

Taxon classificationPlantaePiperalesAristolochiaceae

Y.Fan Wang & Z.R. Guo sp. nov.

BF4BE464-83E3-56B3-B69C-43EE657BCD93

urn:lsid:ipni.org:names:77358999-1

Figs 1 , 2 , 3

#### Type.

China. • Guangdong Province: Zhuhai City, Doumen District, under forest in coastal hills, elevation 90 m, 16 Feb 2024, Y.Fan Wang & Z.R. Guo *yw00036* (holotype: IBSC1041409!; isotypes: IBSC1041410!, IBSC1041411!).

#### Diagnosis.

*Aristolochiazhuhaiensis* shares morphological similarities with *A.thwaitesii*, both of which are distributed in neighboring regions and represent the only two known self-supporting, non-twining Aristolochiasubg.Siphisia species in Asia. In terms of vegetative characteristics, these two congeners are highly similar; however, they can be readily distinguished by their markedly different floral morphologies. *Aristolochiazhuhaiensis* is characterized by a campaniform-infundibuliform calyx limb densely covered with porcelain-white granular pustules. It also features a conspicuous platform-shaped area beneath the perianth throat, which is white, glabrous, waxy, and slightly elevated upward. In contrast, *A.thwaitesii* possesses an elongated, concave tubular calyx limb with an adaxial surface that transitions from purple to pale yellow or white towards the apex. This species is entirely covered with recurved fleshy prickles and lacks the distinctive platform structure found in the calyx of *A.zhuhaiensis*. Detailed morphological comparisons between the new species and *A.thwaitesii* are shown in Figs 4, 5 and Table 1.

**Figure 1.**
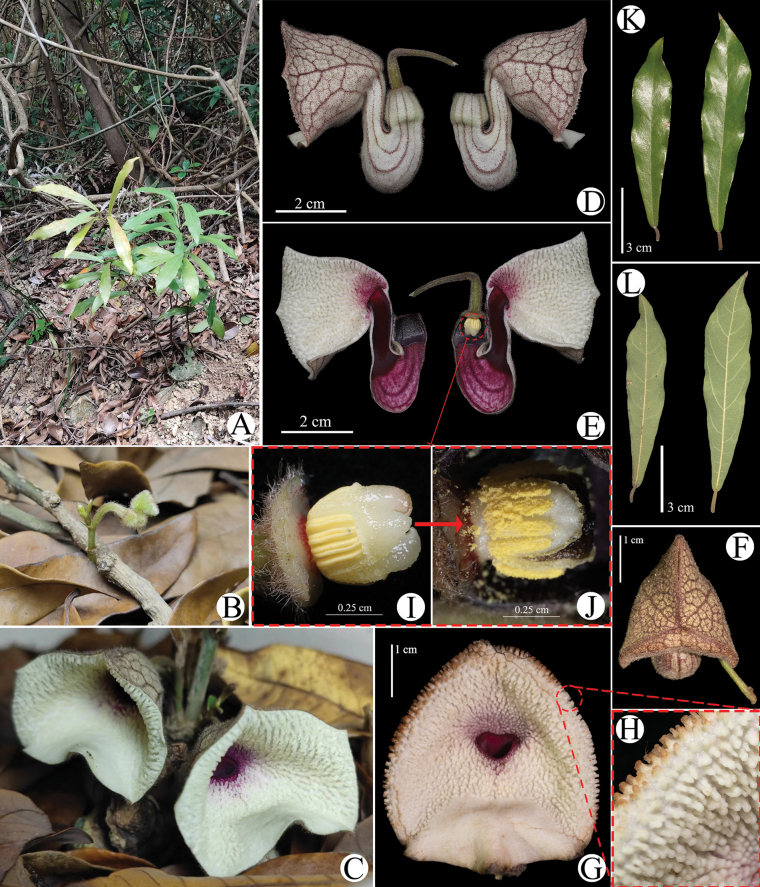
Illustration of *Aristolochiazhuhaiensis***A** habit in situ **B** inflorescence in early development **C** inflorescence **D** lateral view of the flower **E** longitudinal section of the flower **F** front view of the bud **G**, **H** front view of the flower, showing the adaxial side of the calyx limb covered with porcelain-white granular pustules **I**–**J** gynostemium, from female stage (**I**) to male stage (**J**) **K** leaves (adaxial), **L**. Leaves (abaxial). Images **B**–**E**, **I** were provided by Mr. Yiwen Jiang.

**Figure 2.**
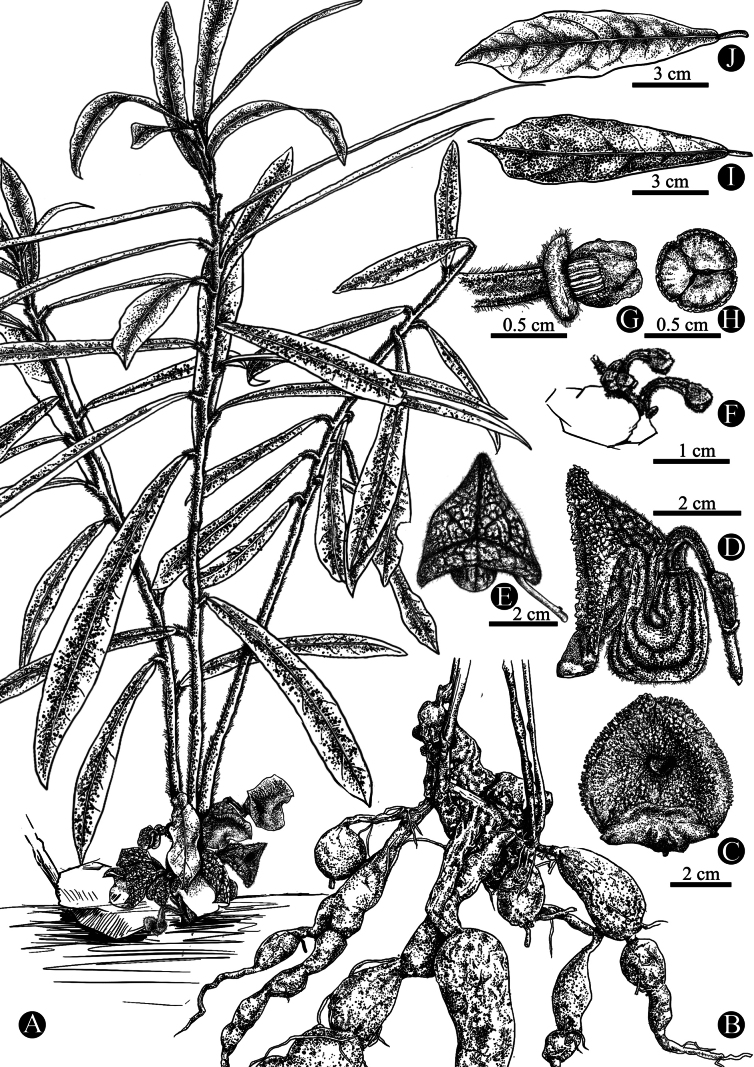
Line drawing of *Aristolochiazhuhaiensis* by Ms. Yushan Cai **A** habit, showing a subshrubby growth form with basal inflorescences **B** roots fusiform or globose **C**, **D** front and lateral view of the flower **E** bud **F** bud sprouts in early development **G**, **H** gynostemium **I** adaxial side of the leaf **J** abaxial view of the leaf, pinnate venation.

**Figure 3.**
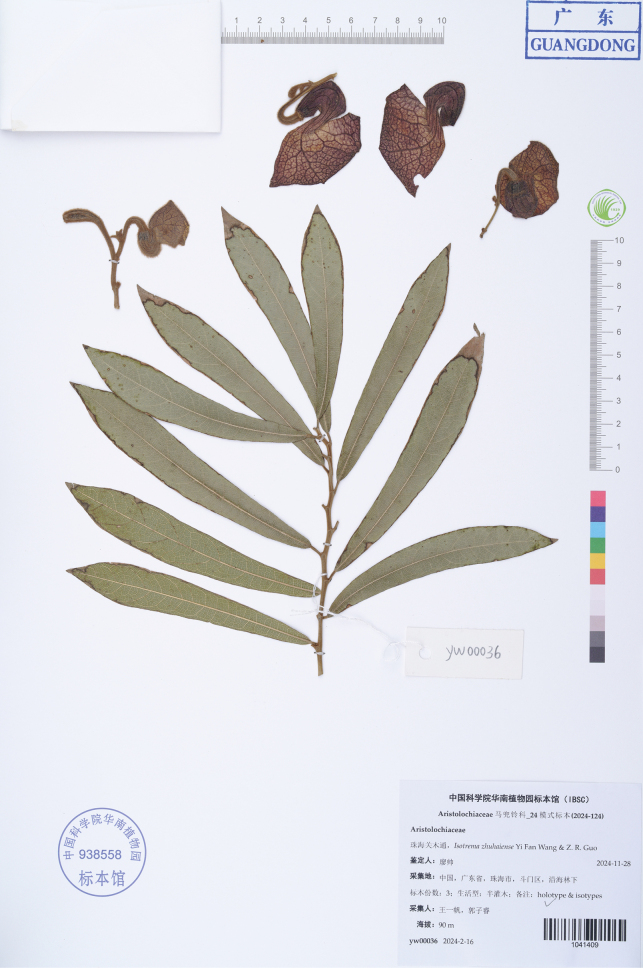
Holotype of *Aristolochiazhuhaiensis* Y.Fan Wang & Z.R. Guo (IBSC 1041409).

**Figure 4.**
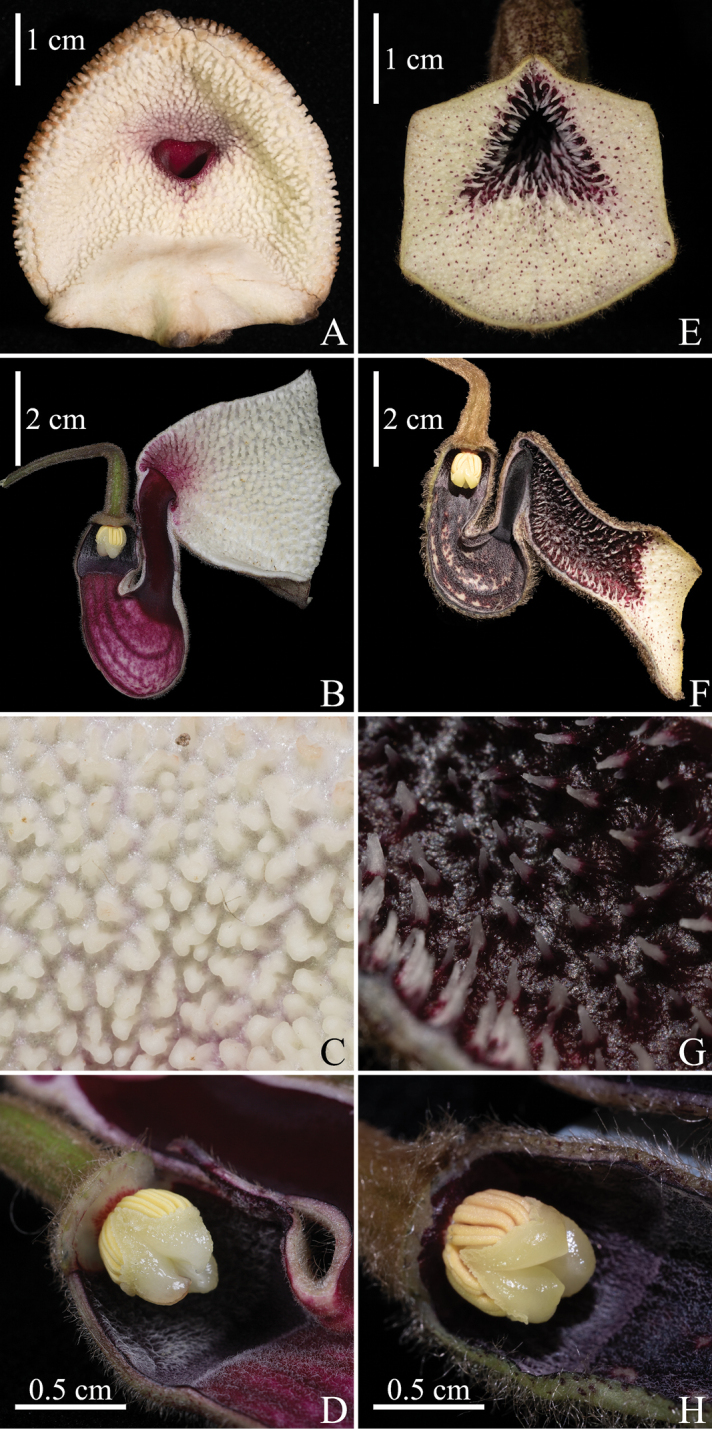
Comparison of the floral morphology of *Aristolochiazhuhaiensis* and *A.thwaitesii***A**–**D***A.zhuhaiensis***A** front view of the flower **B** longitudinal section of the flower **C** porcelain-white granular pustules on adaxial calyx limb **D** gynostemium, stigma apex slightly hooked inward **E–H***A.thwaitesii***E** front view of the flower **F** longitudinal section of the flower **G** recurved purple fleshy prickles on adaxial calyx limb **H** gynostemium with stigma apex not curved.

**Figure 5.**
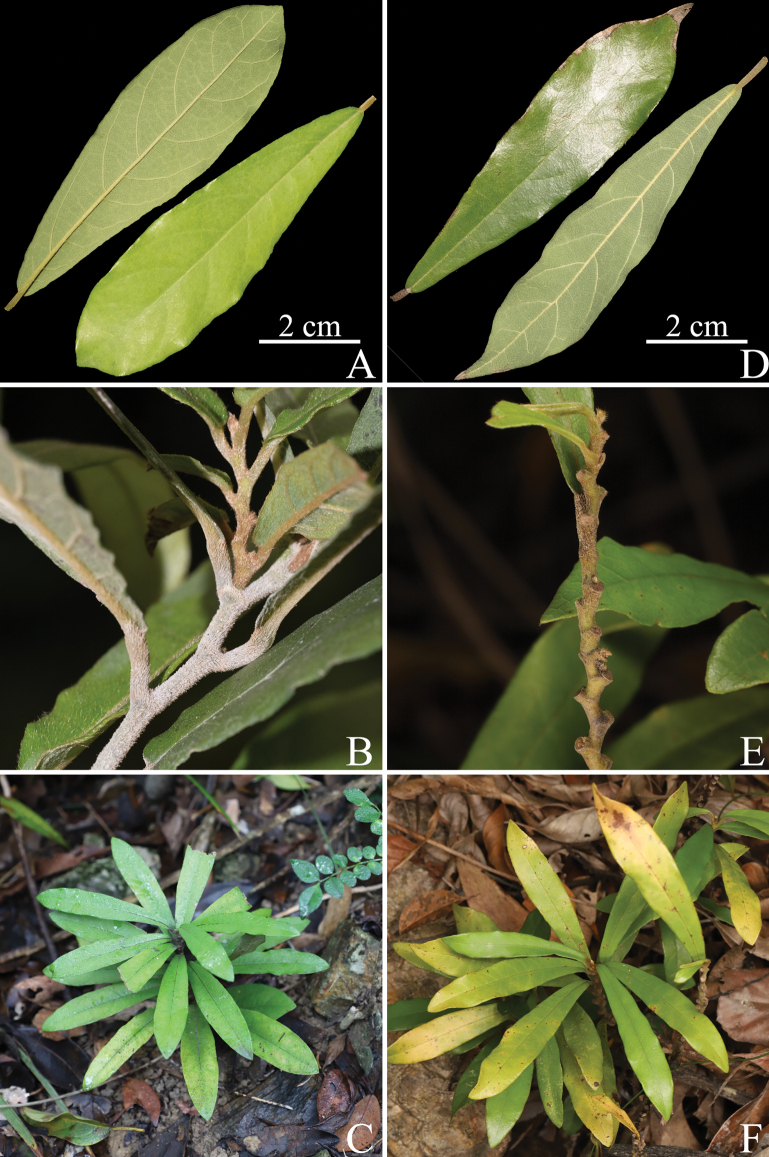
Comparison of the vegetative morphology of *Aristolochiazhuhaiensis* and *A.thwaitesii***A**–**C***A.thwaitesii***A** adaxial (bottom right) and abaxial (top left) side of the leaf **B** petiole and stem **C** habit in situ **D**–**F***A.zhuhaiensis***D** adaxial (top left) and abaxial (bottom right) side of the leaf **E** petiole, nodes and stem **F** habit in situ.

**Table 1.** Morphological comparison and distribution range of *Aristolochiazhuhaiensis* and *A.thwaitesii*.

Characters	*Aristolochiazhuhaiensis*	*Aristolochiathwaitesii*
**Distribution**	Zhuhai, Guangdong, China	Hong Kong SAR, China
**Habit**	Erect subshrub	Erect subshrub
**Leaf**		
**shape**	Spathulate, narrowly oblanceolate, or lanceolate, with an apex acute to acuminate	Spathulate, narrowly oblanceolate, or oblong-oblanceolate, with an apex acuminate to obtuse
**size**	12–16 × 2–2.5 cm	10–15 × 2.5–3 cm
**pubescence (adaxial side)**	Almost glabrous to subglabrous adaxially, densely brown-villous abaxially	Subglabrous adaxially, densely brown-villous abaxially
**venation**	Pinnate, 10–12 secondary veins each side	Pinnate, 10–12 secondary veins each side
**Inflorescence**		
**inflorescence**	Basal inflorescence; flowers in dense fascicles; fascicles with variable flower count	Basal inflorescence; flowers in dense fascicles; fascicles with variable flower count
**flower count**	Solitary to 20 flowers	Solitary to 30–40 flowers
**Perianth**		
**calyx utricle (adaxial side)**	Campanulate, 0.9–1.2 × 0.8–1.0 cm; dark purplish, arachnoid white villous	Cylindric to spherical, 0.8–1.0 × 0.6–0.8 cm; dark purplish, arachnoid white villous
**calyx tube (adaxial side, U–G–T: utricle to geniculation to throat)**	U–G: 2.5–3.5 cm, light purplish with dark veins, subglabrous; G–T: 2.5–3.0 cm, dark purple, glabrous, velvety	U–G: 1.8–2.8 cm, purplish with white patches, subglabrous, slightly ridged with veins; G–T: 2.0–2.5 cm, dark purple to almost black, glabrous, velvety
**calyx limb (adaxial side)**	Calyx limb 3.0–4.0 cm long, 4.5–5.5 cm in diameter, flaring to campaniform-infundibuliform; densely covered with porcelain-white granular pustules; beneath the throat forms a glabrous and unarmed raised platform	Calyx limb elongate to concave tubular shape, 3.5–4.0 cm long, 2.0–2.5 cm in diameter; fully covered with recurved fleshy prickles; Calyx margin white to pale yellow, 3 dentate; dark purple inside tubular calyx limb without any platform-like Structure
**calyx throat**	Throat ca. 6–9 mm in diameter; purple, cordate-shaped	Throat ca. 5–8 mm in diameter, purple to black; elliptic-shaped
**Gynostemium**	Lobe apex hooked inward	Lobe apex obtuse

#### Description.

Erect subshrub. Roots fusiform or globose. Stems terete, densely rusty villous, becoming glabrous when lignified. Leaf blades spathulate, narrowly oblanceolate, or lanceolate, with an apex acute to acuminate, 12–16 × 3–3.5 cm. Almost glabrous to subglabrous adaxially, densely brown-villous abaxially. Venation pinnate, with 10–12 secondary veins on each side, leaves papery; petiole approximately 0.6–1.0 cm long, densely rusty villous. Basal inflorescence, fasciculate; flower count varies significantly depending on plant nutrient status, ranging from solitary to up to 20. Pedicels approximately 1–1.2 cm long, initially erect, then pendulous, densely rusty villous. Bracteole 1, lanceolate, 1–2 × 0.4–0.5 mm, densely rusty villous on both sides, sessile. Perianth zygomorphic, densely covered with grey indumentum abaxially, campanulate, 0.9–1.2 × 0.8–1.0 cm; dark purplish, arachnoid white villous. Calyx tube geniculately curved, yellowish to brownish abaxially; utricle to geniculation: 2.5–3.5 cm, light purplish with dark veins, subglabrous adaxially; geniculation to throat: 2.5–3.0 cm, dark purple, glabrous, velvety adaxially. Calyx limb 3.0–4.0 cm long, 4.5–5.5 cm in diameter, flaring to campaniform-infundibuliform, densely covered with porcelain-white granular pustules; beneath the throat forms a glabrous and flat area that lies on the soil, waxy; abaxial side of calyx limb brownish to yellowish, villous with ridged veins. Apex of the calyx limb 3-dentate; when the flower is near withering, the calyx apex rolls outward. Throat approximately 6–9 mm in diameter, purple, cordate-shaped. Stamens 6 in one series, fully adnate in 3 pairs to the style column to form a gynostemium, opposite the stigma lobes; anthers oblong, approximately 2.0–3.0 mm long, extrorse. Gynostemium approximately 4.5–5 mm long, 4–5 mm in diameter, fleshy; lobes 3, hooked inward at the apex; margin crenate-rugose. Ovary inferior, cylindric, 6–8 mm long, 3–4 mm in diameter, densely rusty villous abaxially.

#### Distribution and habitat.

**Figure 6.**
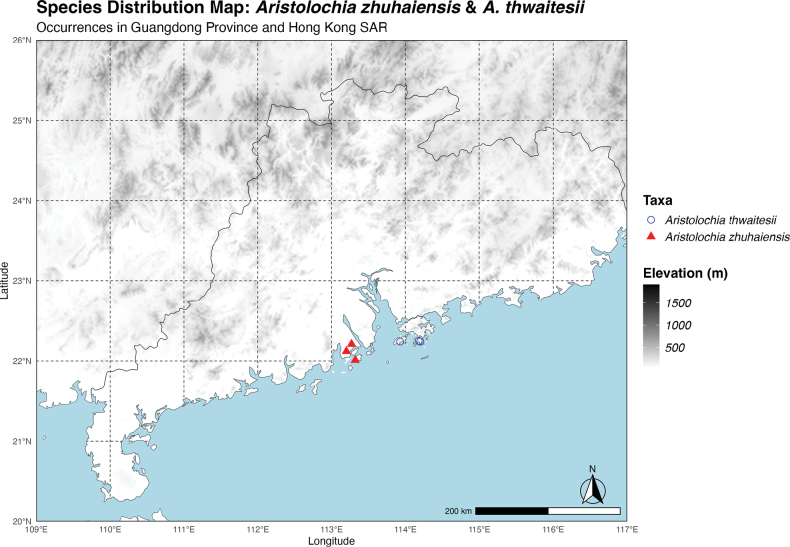
Distribution map of *Aristolochiazhuhaiensis* Y.Fan Wang & Z.R. Guo, sp. nov., highlighting its geographically close yet isolated distribution, with *A.thwaitesii* primarily found in Hong Kong SAR and *A.zhuhaiensis* observed in Zhuhai City.

The new species has been discovered exclusively in Zhuhai City, Guangdong Province, China (Fig. 6). It is typically found near creeks within forests on coastal hill slopes, at elevations ranging from 50 to 100 meters. To date, three populations have been identified, all situated in the southern region of Zhuhai City: two within Doumen District and one in Jinwan District, each in proximity to the coastal areas. This species commonly occurs near water sources such as creeks and mountain streams, usually thriving under dense canopy cover. Associated species typically include *Nepenthesmirabilis* (Lour.) Druce, *Droseraspatulata* Labill., *Strophanthusdivaricatus* (Lour.) Hook. & Arn., *Pterisvittata* L., and *Rhodomyrtustomentosa* (Aiton) Hassk.

#### Phenology.

Flowering from January to early March, fruit or seed not seen.

#### Etymology.

The epithet ‘*zhuhaiensis*’ refers to the type locality (Zhuhai City), where the new species has been discovered. Given Zhuhai’s status as a highly populated and urbanized city, the discovery of a new species is both a rare and welcome occurrence.

#### Vernacular name.

During our initial encounter with this new species, local cultivators referred to it by the Cantonese vernacular name “shí qín qú” (石蠄蟝), indicating that the root, harvested for medicinal purposes, has a surface texture so coarse it resembles a toad’s skin. Interestingly, as our survey extended further, we discovered that the Hakka people refer to the species as “shù zǎi lián” (树仔莲), which translates literally to “shrubby herb medicine.” These two distinct ethnic groups each have their own vernacular name for the same species. For clarity and consistency in scientific communication with the broader public, we propose the Chinese name “zhū hǎi guān mù tōng” (珠海关木通), reflecting the literal meaning of its epithet and its geographical origin.

#### Conservation status.

Since we first discovered and recognized it as a new species, we have conducted more than five extensive field trips covering the coastal mountain hills, including all major coastal cities within the Greater Bay Area, particularly focusing on Zhuhai, and its neighboring cities Jiangmen and Shenzhen. Despite these efforts, we only located three extant populations, all of which are confined to Zhuhai. As previously mentioned, this species has long been recognized by local medicinal practitioners and has been exploited for generations to treat snake bites, according to local testimony. The traditional belief in its medicinal properties has made this species highly sought after, leading to its scarcity in the wild in recent years.

Our investigation revealed that *A.zhuhaiensis* is morphologically challenging to distinguish from its close congener, *A.thwaitesii*, when not in flower. Local collectors report sometimes to travel to outlying islands near Hong Kong or the Pearl River Estuary to harvest this plant. We hypothesize that in these cases, it remains unclear whether the collected individuals are *A.zhuhaiensis* or *A.thwaitesii*, especially as both species primarily exhibit vegetative growth for much of the year, and the collectors focus mainly on harvesting the tuberous roots based on our field survey.

*Aristolochiazhuhaiensis* faces several severe threats. While *A.thwaitesii* is already classified as Vulnerable (VU) according to the China Plant Specialist Group (2019), the conservation status of *A.zhuhaiensis* appears to be even more precarious. The three populations we identified are situated on very small hills in close proximity to urban areas. These hills have limited area and gentle slopes and are completely encircled by urban developments, including residential communities, real estate projects, tourist attractions, airport, and farmlands, as well as other anthropogenic landscapes. Furthermore, these habitats have been heavily invaded by artificial fast-growing *Eucalyptus* plantations. Our field observations indicate that *A.zhuhaiensis* exclusively grows under native coastal low shrubland along riparian zones and does not thrive in secondary forests or plantation environments. Habitat loss due to its proximity to human activity is likely one of the primary threats to its survival.

During our multiple fieldwork expeditions in the region, we observed a substantial number of flowers produced by robust individuals, with some bearing up to 20 flowers in a single fascicle at the basal stem. However, we found no evidence of fruiting or seed production. The basal flowering habit might indicate specific pollinator requirements, a topic that warrants further ecological study.

Another aspect of the potential ecological significance of *A.zhuhaiensis* is its possible role as a host plant for the Chinese windmill butterfly *Byasaalcinous* Klug, which is also associated with *A.thwaitesii*. This host relationship with *A.thwaitesii* has been confirmed by the Agriculture, Fisheries and Conservation Department (AFCD) of the Hong Kong SAR (Anon 2010). Considering the morphological similarities between *A.zhuhaiensis* and *A.thwaitesii*, as well as the natural range of *B.alcinous* extending into Zhuhai (Wu and Bai 2001), *A.zhuhaiensis* could also provide a vital food source for the butterfly. Further ecological studies are needed to confirm this potential interaction.

Currently, the three known populations of *A.zhuhaiensis* are located in the coastal region of Zhuhai City, each in close proximity and restricted to an area of less than 10 km^2^, at elevations ranging from 50 to 100 meters. Based on these populations, the estimated extent of occurrence (EOO) is 0.335 km^2^ (<100 km^2^), and the area of occupancy (AOO) is 12.000 km^2^. No fruiting or seed production has been observed over the past two consecutive years, and each population comprises fewer than ten individuals. Based on these findings, we recommend classifying *A.zhuhaiensis* as Critically Endangered (CR) under the IUCN Red List criteria B1ab(i,ii,iii,iv,v); D1. This recommendation reflects its extremely limited distribution, ecological significance, close association with urban areas, vulnerability to habitat loss and medicinal exploitation, and the absence of observed sexual reproduction.

#### Additional specimen of *Aristolochiazhuhaiensis* examined.

China. • Guangdong Province: Zhuhai City, Doumen District, under forest in coastal hills, elevation 68 m, 2 Mar 2024, Y.Fan Wang & Z.R. Guo *yw00077* (paratype: IBSC1041412!).

#### Specimens of *Aristolochiathwaitesii* examined.

China. • Hongkong: Wong Nai Chung Gap, 1903, W.J. Tutcher *979* (IBSC0127925), 4 Jul 2000, Y.W. Lam *1633* (HK38320); Lantau Island, 16 Mar 1909, W.J. Tutcher *8273* (IBSC0127926 & HK23774). UK. Cultivated at Kew, Royal Botanic Gardens, *s.coll. s.n.*, April 1858 (K003691508); • Tze Kong Bridge, Repulse Bay, Southern District, Hong Kong Island, 1 April 2024 Y.Fan Wang *yw00068* (IBSC!); Tze Kong Bridge, Repulse Bay, Southern District, Hong Kong Island, 2 April 2024 Y.Fan Wang *yw00069* (IBSC!).

#### Notes.

*Aristolochiathwaitesii* has been confirmed, by our fieldwork, to occur in the Hong Kong region, specifically on Hong Kong Island and Lantau Island (Fig. 6). Although there are records indicating its discovery in Jiangmen and Zhuhai (Ma 1989; Huang et al. 2003), no living plant collection, or photograph of the flower has provided conclusive evidence of its presence there. The only two voucher specimens (IBSC0127924 & SYS00159983), collected in 1973 and 1979, almost 50 years ago, are solely vegetative specimens, lacking flowers. Following the confirmation of *A.zhuhaiensis*, we re-examined these two voucher specimens and embarked on a field expedition in early July 2024 to Dawanshan Island, where voucher IBSC0127924 was originally collected. Despite extensive efforts, we failed to locate any *Aristolochia* individuals. Administratively, Wanshan Island is under Zhuhai’s jurisdiction, but geographically it lies closer to the known distribution of *A.thwaitesii* in Hong Kong than to the range of *A.zhuhaiensis* that we have documented. As for voucher SYS00159983, despite multiple extensive field surveys in Jiangmen City, no specimens have been found. Given the current lack of definitive evidence, such as a living specimen or a photograph confirming the floral morphology, we have refrained from assigning either voucher to *A.thwaitesii* or *A.zhuhaiensis* until further conclusive data can clarify their identity.

Given the proximity of this species to urban areas and the lack of any form of protection or monitoring in its natural habitats, combined with its long history of medicinal exploitation, we have opted not to disclose the precise locations of these populations in this paper to safeguard their conservation. We strongly urge local authorities to implement policies or legislation to protect this rare species. Researchers requiring material or information for scientific purposes may contact the authors directly for collaboration and information sharing.

### ﻿Key to *Aristolochiazhuhaiensis* and morphologically close species

**Table:** 

1	Habit erect subshrub	**2**
–	Habit climbing shrub or woody liana	**3**
2	Calyx limb campaniform-infundibuliform, densely covered with porcelain-white granular pustules; platform-shaped area beneath perianth throat present	***A.zhuhaiensis***
–	Calyx limb elongated, concave tubular, adaxially transitioning from purple to pale yellow or white; covered with recurved fleshy prickles; without granular pustules or platform structure	***A.thwaitesii***
3	Leaf elliptic or ovate, apex obtuse or slightly mucronate; older lignified stems forming broad corky wings on the bark	***A.fangchi***
–	Leaf elliptic, oblanceolate, or ovate, apex acuminate; older lignified stems with a fissured surface	**4**
4	Calyx limb spreading, throat exposed; perianth adaxial surface smooth	***A.westlandii***
–	Calyx limb connate, forming a crateriform or tubular structure encircling the throat	**5**
5	Calyx limb connate into a tubular structure, tilted downward, throat not visible externally	***A.plagiostoma***
–	Calyx limb connate into a crateriform, throat visible from a frontal view	**6**
6	Basal portion of calyx limb shorter than or roughly equal to the upper; limb adaxially yellow with dark purple stripes	***A.pilosistyla***
–	Basal portion of calyx limb longer than the upper; limb adaxially dark purple, with a bright yellow throat	***A.championii***

## Supplementary Material

XML Treatment for Aristolochia zhuhaiensis

## ﻿Additional information

### Author ORCIDs

Yi-Fan Wang https://orcid.org/0009-0003-1339-1856

Zi-Rui Guo https://orcid.org/0009-0004-1817-9827

Sven Landrein https://orcid.org/0000-0003-0028-2450

Joyce G. Onyenedum https://orcid.org/0000-0002-1047-9807

Shuai Liao https://orcid.org/0000-0002-3876-8002

### Data availability

All of the data that support the findings of this study are available in the main text.
